# Supplementary material for: Reconstitution of surface lipoprotein translocation through the Slam translocon
Source: eLife. 2022 Apr 27;11:e72822. doi: 10.7554/eLife.72822 (PMC9090332; doi:10.7554/eLife.72822)
Supplement: Supplementary file 1. — Raw images for figures and figure supplements. [file elife-72822-supp1.docx]

**Supplementary File 1**

**Material Supplementary Table**: Strains, plasmids and antibodies used in this study.

| **Strain or plasmid** | **Description** | **Reference** |
| --- | --- | --- |
| **Strains:** |  |  |
| *E. coli MM294* | *F-, glnX44(AS), LAM-, rfbC1, endA1, spoT1, thiE1, hsdR17, creC510* | Yale CGSC #6315 |
| *E. coli C43 (DE3)* | *F– ompT gal dcm hsdSB(rB- mB-)(DE3)* | (26) |
| *E. coli BL21 Star (DE3)* | *F– ompT gal dcm hsdSB(rB- mB-)gal dcm rne131(DE3)* | Invitrogen, C6010-03 |
| *E. coli* K12 | F´proA+B+ lacIq Δ(lacZ)M15 zzf::Tn10(TetR)/ fhuA2 glnV Δ(lac-proAB) thi-1 Δ(hsdS-mcrB)5 | (26) |
| *E. coli* K12 *skp-* | *E. coli* K12 devoid function of Skp | (26) |
| *E. coli* K12 *degp-* | *E. coli* K12 devoid function of DegP | (26) |
| *N. meningitidis* B16B6 |  | (1) |
| *N. meningitidis* B16B6 *Δslam1* | *Δslam1::kan* | (11) |
| *N. meningitidis* B16B6 *ΔtbpB* | *ΔtbpB::kan* | (11) |
| *N. meningitidis* B16B6 *Δskp* | *Δskp::kan* | This study |
| **Plasmids:** |  |  |
| pET26b | *E. coli* expression vector used for C43 (DE3) strain | Addgene, 69862-3 |
| pET26 *Nme* Slam1 | pET26 containing pelB signal peptide, 6xHis and mature *N. meningitidis* Slam1 | (11) |
| pET26 *Mcat* Slam1 | pET26 containing pelB signal peptide, 6xHis and mature *M. catarrhalis* Slam1 | This study |
| pET26 *Nme* Slam2 | pET26 containing pelB signal peptide, 6xHis and mature *N. meningitidis* Slam2 | This study |
| pET26 *Ngo* Slam2 | pET26 containing pelB signal peptide, 6xHis and mature *N. gonorrhoeae* Slam2 | This study |
| pET52b | Expression vector used for *E. coli* C43 (DE3) in this study | Novagen, 71554-3 |
| pET52 *Mcat* TbpB-flag | pET52b containing full-length *M. catarrhalis* TbpB with and a flag tag | This study |
| pET52 *Nme* HpuA | pET52b containing full-length *N. meningitidis* HpuA | This study |
| pET52 *Nme* HpuA-flag | pET52b containing full length *N. meningitidis* HpuA and a flag tag | This study |
| pET28a | Expression vector used for BL21 (DE3) strain in this study | Novagen, 69864-3 |
| pET28 LolA | pET28a containing 6xHis tag and mature *E. coli* LolA | This study |
| pET28 *E. coli* SurA | pET28a containing 6xHis tag and mature *E. coli* SurA | This study |
| pET28 *E. coli* Skp | pET28a containing 6xHis tag and mature *E. coli* Skp | This study |
| pET28 *E. coli* DegP | pET28a containing 6xHis tag and mature *E. coli* DegP | This study |
| pET28 *Nme* Skp | pET28a containing 6xHis tag and mature *N. meningitidis* Skp | This study |
| pJH114 | pTRC99a containing *E. coli* BamA-E with an 8xHis tag on the C-terminus of BamE obtained from Dr. Harry Bernstein | (16) |
| pGCC4 | Expression vector used for *E. coli* K12 strain in this study | Addgene,  37058 |
| pGCC4 Mcat Slam1 | pGCC4 containing 6xHis tag and mature *M. catarrhalis* Slam1 | This study |
| pHERD | Expression vector used for *E. coli* K12 strain in this study | NovoPro, V005568 |
| pHERD Mcat TbpB-flag | pHERD containing mature *M. catarrhalis* TbpB C-terminal flag-tag | This study |
| pUC19 | Cloning vector for bacteria | Addgene, 50005 |
| pUC19 *skp ±* 500 | pUC19 containing *N. meningitidis skp* gene with 500bp DNA upstream and downstream of *skp* | This study |
| pUC19 Δ*skp::kan* ±500 | pUC19 containing *kan* gene with 500bp DNA upstream and downstream of *skp* (*skp* gen is replaced/swapped by *kan* gene) | This study |
| pGCC4 Nme Skp | pGCC4 plasmid containing *skp* gene (with its endogenous signal peptide) | This study |
|  |  |  |
| **Antibodies:** |  |  |
| α -Flag | Rabbit polyclonal antibody for Flag (DYKDDDDK) epitope | Invitrogen, PA1-984B |
| α -OmpA | Rabbit polyclonal antibodies were obtained from Dr. Jan Willem de Gier | (43) |
| α-His | Mouse monoclonal antibody for detecting polyhistidine tag | Pierce, MA1213 |
| α-TbpB | Rabbit polyclonal antibodies against *N. meningitidis* B16B6 TbpB | (11) |
| α-Skp (*E. coli)* | Mouse polyclonal antibody for detecting *E. coli* Skp | This study |
| α-Skp (*Nme)* | Mouse polyclonal antibody for detecting *N. meningitidis* Skp | This study |
| HRP-rabbit IgG | Goat anti-rabbit IgG antibody conjugated to horseradish peroxidase (HRP) | Cell Signalling, #7074S |
| HRP-mouse IgG | Goat anti-mouse IgG antibody conjugated to horseradish peroxidase (HRP) | Pierce, PI31430 |
| Strep-HRP | Streptavidin conjugated to horseradish peroxidase (HRP) | Thermo Scientific™, N100 |
| Strep-PE | Streptavidin conjugated to phycoerythrin (PE) | Jackson&ImmunoResearch, 016-110-084 |
| Rabbit-PE | Goat anti-rabbit IgG conjugated to phycoerythrin (PE) | Jackson&ImmunoResearch, 111-116-144 |
|  |  |  |
| **Substrate:** |  |  |
| Bio-htf | Biotinylated human transferrin | Invitrogen™, T23363 |
